# Patterns of thyroid hormone levels in pediatric medullary thyroid carcinoma patients on vandetanib therapy

**DOI:** 10.1186/1687-9856-2015-3

**Published:** 2015-02-16

**Authors:** Maya Lodish, Alexandra Gkourogianni, Ethan Bornstein, Ninet Sinaii, Elizabeth Fox, Meredith Chuk, Leigh Marcus, Srivandana Akshintala, Frank Balis, Brigitte Widemann, Constantine A Stratakis

**Affiliations:** National Institute of Child Health & Human Development, National Institutes of Health (NIH), Building 10-CRC, room 1-3330 10 Center Drive, Bethesda, MD 20892 USA; Epidemiology & Biostatistics, CC, NIH BG 10 RM 2 N228 10 Center Drive, Bethesda, MD 20814 USA; The Children’s Hospital of Philadelphia, Colket Translational Research Building 4016, 3501 Civic Center Blvd, Philadelphia, PA 19104 USA; Pediatric Oncology Branch, National Cancer Institute, NIH BG 10-CRC RM 1-5750 10 Center Dr., Bethesda, MD 20814 USA

**Keywords:** Multiple Endocrine Neoplasia type 2 B (MEN2B), Medullary thyroid carcinoma (MTC), Tyrosine kinase inhibitor (TKIs), Vandetanib

## Abstract

**Background:**

Tyrosine kinase inhibitors (TKIs) have been associated with elevated TSH as a drug class effect. Prior studies of vandetanib in adults with medullary thyroid carcinoma (MTC) described an increase in levothyroxine (LT) requirement. We studied TSH, free T4, and LT dosing in children and adolescents enrolled in the phase I/II trial of vandetanib for medullary thyroid cancer (MTC)

**Methods:**

Data from 13 patients with multiple endocrine neoplasia type 2B (MEN 2B) and MTC were analyzed [6 M, 7 F, median age 13.0 y (9.1-17.3)] Eleven patients (85%) had undergone prior thyroidectomy and all received single-drug therapy with vandetanib for > 6 months. Confirmed compliance with vandetanib (67–150 mg/m^2^/day) and LT was a necessary inclusion criterion.

**Results:**

While on vandetanib treatment, all 11 athyerotic patients exhibited significantly increased TSH levels. The baseline TSH level was 4.37 mclU/ml (0.08 - 23.30); in comparison, the first peak TSH concentration on vandetanib was 15.70 mclU/ml (12.50 - 137.00, p = 0.0010). The median time to reach the initial peak of elevated TSH was 1.8 months (0.3 - 9.3). Free T4 levels remained within the normal reference range. An increase from a baseline LT dose of 91 mcg/m^2^/day (±24) to 116 mcg/m^2^/day (±24) was required in order to resume normative TSH levels (p = 0.00005), equal to an increase of 36.6% (±16.56) in the dosage of LT in mcg/day. For the 2 patients with intact thyroid glands, free T4 and TSH remained normal over a combined 6 patient years of follow up.

**Conclusions:**

In our cohort of pediatric MTC patients, athyreotic patients with preexisting hypothyroidism developed increased TSH and reduced free T4 during the first few months of treatment with vandetanib, necessitating an increase in LT dosage. Additional patients with normal thyroid function before treatment and intact glands (n = 2) maintained normal thyroid function tests during treatment. Elevated TSH in athyreotic patients may be due to an indirect effect of vandetanib on the metabolism of thyroid hormone, or to altered TSH sensitivity at the pituitary. Proper recognition and management of abnormal thyroid hormone levels is critical in growing children on TKIs.

**Trial registration:**

ClinicalTrials.gov Identifier: NCT00514046

## Background

Hereditary medullary thyroid carcinoma (MTC) is a manifestation of multiple endocrine neoplasia (MEN) type 2A and MEN 2B syndromes caused by germline, activating mutations in the RET (REarranged during Transfection) proto-oncogene (10q11.2). MTC accounts for 5-10% of all pediatric thyroid malignancies and its annual incidence is 0.3-2 cases per 10^6^ children (1 M: 1 F) [1–3].

Only 25% of MTC cases are hereditary (Familial MTC (FMTC), MEN2A, MEN2B) while the majority, 75% of MTC cases, are sporadic. Hereditary MTC is multifocal, bilateral, indolent and usually presents with metastasis at the time of diagnosis. If diagnosed while the tumor is confined to the thyroid, MTC has a favorable prognosis (10 year survival rate 70-80%) [4]. Common sites of metastatic disease include cervical and mediastinal lymph nodes, as well as lungs, liver, and bone; in the case of metastatic disease the 10 year survival rate drops to approximately 40% [4–7]. Metastatic or locally advanced MTC is unresponsive to cytotoxic chemotherapy or radiation [4, 8]. Targeted therapy with Tyrosine Kinase inhibitors (TKIs) are now approved to treat progressive and advanced MTC. TKIs compete with the ATP-binding domains of the TK catalytic unit inhibiting the activation of oncogenic intracellular signaling pathways [9]. Vandetanib (Caprelsa, Astra Zeneca Pharmaceuticals) is an orally bioavailable multi-RTK (receptor Tyrosine kinase) inhibitor that blocks the mutant RET gene product and has anti-tumor activity in adults with hereditary MTC [10, 11]. At the National Cancer Institute, a phase I/II trial of vandetanib for children and adolescents with MTC was conducted to define a recommended dose and assess antitumor activity, as described by Fox et al. [12]. A common non-dose limiting toxicity was TSH elevation necessitating an increase in levothyroxine (LT) dosage – however, this was only noted in athyreotic patients who were previously on a stable dose. TKIs have been associated with elevated TSH as a drug class effect [13]; two prior studies of vandetanib in adults with MTC described an increase in LT requirement [14, 15]. Little is known about the effect of vandetanib on thyroid hormone levels in children. Our objective was to describe the TSH and free T4 levels and LT dosing in children and adolescents enrolled in the phase I/II trial of vandetanib for Multiple Endocrine Neoplasia Type 2B (MEN 2B) and MTC, NCT00514046.

## Methods

Data from 13 patients with MEN 2B and MTC were analyzed [6 M, 7 F, median age 13.0 y (9.1-17.3)] (Table 1). Patients were enrolled in the phase I/II trial of vandetanib for Multiple Endocrine Neoplasia Type 2B (MEN 2B) and MTC, NCT00514046. Informed consent from the patients’ parents (and assent from older children) was obtained for all patients. Eleven patients (85%) had undergone prior thyroidectomy and all received single-drug therapy with vandetanib for > 6 months. Confirmed compliance with vandetanib (67–150 mg/m^2^/day) and LT was a necessary inclusion criterion (Table 1). Patients were instructed to take LT on an empty stomach and not with other medications or calcium/soy containing products. One patient was excluded due to non-compliance with his replacement with LT. Data were analyzed using paired t-tests for normally distributed data and the Wilcoxon signed rank test for non-parametric data, and are reported as mean (±SD) or median (range).

**Table 1:** **Patient Characteristics**

Number of patients	13
Gender (male/female)	6/7
Age at start of enrollment on clinical trial (median, range)	13.0 years (9.1-17.3)
RET mutation	M918T (n = 13)
Baseline LT dose	100 mcg/day (57–200)

## Results

**Figure 1:**
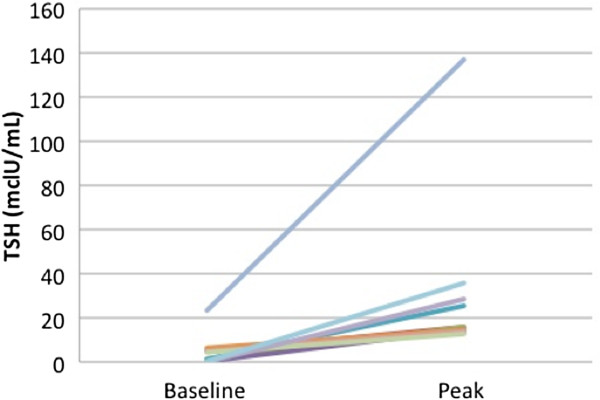
**TSH levels prior to initiation of vandetanib in thyroidectomized patients compared to first peak TSH concentration on study p = 0.0010.**

**Figure 2:**
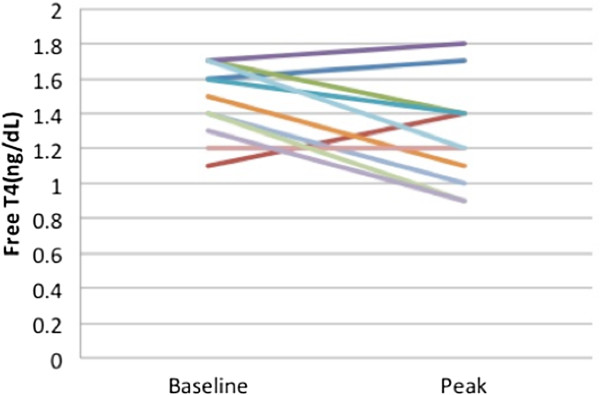
**Free T4 levels prior to initiation of vandetanib in thyroidectomized patients compared to levels at first peak TSH concentration, p = 0.039.**

**Figure 3:**
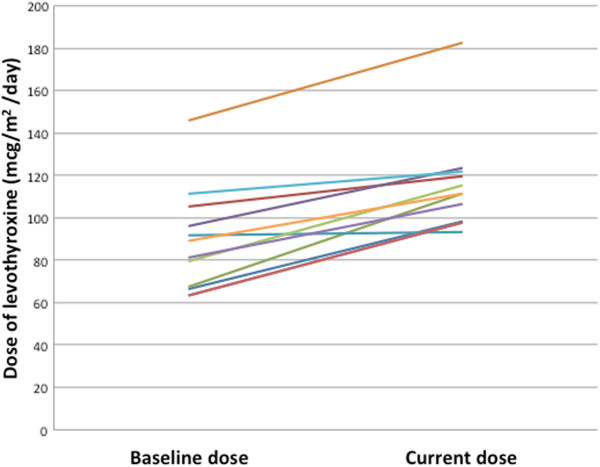
**Dose of LT (mcg/m** ^**2**^ **/day at baseline vs. dose required in order to resume normative TSH in thyroidectomized patients, p = (p = 0.00005).**

While on vandetanib treatment, all 11 athyreotic patients exhibited significantly increased TSH levels. The baseline TSH level was 4.37 mclU/ml (0.08 - 23.30). In comparison, the first peak TSH concentration on vandetanib was 15.70 mclU/ml (12.50 - 137.00, p = 0.0010) (Figure 1). The median time it took to reach the first peak of elevated TSH was 1.8 months (0.3 - 9.3). Free T4 levels remained within the normal reference range, yet significantly decreased from baseline levels of 1.47 ng/dL (±0.21) to 1.27 ng/dL (±0.30) when measured at the time of maximum TSH (p = 0.039) (Figure 2). TSH levels normalized after subsequent increases in LT dose. An increase from a baseline LT dose of 91 mcg/m^2^/day (±24) to 116 mcg/m^2^/day (±24) was required in order to resume normative TSH levels (p = 0.00005), (Figure 3), equal to an increase of 36.6% (±16.56) in the dosage of LT in mcg/day. There were no clinical sequelae as a result of the elevations in TSH. Thyroid hormone levels increased, either due to a change in LT dose, or in one patient, due to vandetanib being held for oral surgery without adjustment of the LT dose to account for the effect of the TKI. Linear growth was closely monitored for the duration of the study. The median percentile of height for age at baseline was 30 (<3-96)%, and increased to 55 (<3-96)% at the last evaluation (P = 0.03). The median percentile of weight for age at baseline was 9 (<3-96)% and increased to 20 (<3-91)% at last evaluation (P = 0.48). For the 2 patients with thyroid glands still intact, free T4 averaged 1.17 ± 0.15, (normal range 0.8-1.5 ng/dL) and TSH levels averaged 3.48 ± 2.18, (normal range 0.4-4 mclU/ml) over a combined 6 patient years of follow up.

## Discussion

Innovative therapeutic agents that target genetic alterations (selective cancer therapy) associated with the development of MTC seem promising to treat progressive and advanced MTC [16]. Even if TKIs combine a high therapeutic window with less toxicity than conventional chemotherapy, vandetanib’s adverse effects varied in respect to the administered dose and included rash, nausea, hypertension, headache, ECG QTc prolongation and endocrine effects [17]. In a randomized controlled trial of vandetanib for adults with MTC in which 90% of patients enrolled had prior thyroidectomy, increased dosing of LT was required in 49.3% of vandetanib treated patients compared to 17.2% of placebo-treated patients [14]. In our cohort of pediatric MTC patients, patients who were rendered hypothyroid after thyroidectomy developed increased TSH and reduced free T4 during the first few months of treatment with vandetanib, necessitating an increase in LT dosage. Over the duration of this ongoing study, we continue to adjust LT dosing individualized to each patient as dosages of TKIs are altered, and patients grow and progress through puberty. Interestingly, while on vandetanib, 2 patients with normal pretreatment thyroid functions and intact glands continued to have TFTs within the normal range without requiring thyroid hormone treatment. Several studies report that TKIs either induce recurrence of hypothyroidism in hypothyroid patients, increasing their needs in LT replacement, or induce hypothyroidism in patients with previously normal thyroid function [14, 18–20]. In a study looking at endocrine function in 35 adults with thyroid cancer on vandetanib, LT dose had to be increased for 26 patients, 5 with differentiated thyroid cancer and 21 with MTC [15]. Similar to our findings with children, none of the three adult nonthyroidectomized MTC patients needed LT treatment during the study according to normal serial TSH levels on vandetanib [15]. Elevated TSH in athyreotic patients may be due to an indirect effect of vandetanib on the metabolism of thyroid hormone, or with thyroid hormone action at the pituitary level. Impaired type-2 iodothyronine deiodinase and reduction of T3 generation from T4 at the pituitary level has been proposed as one alternative mechanism contributing to the inappropriate elevation of TSH in patients on TKI, although direct evidence is lacking [21]. Another hypothesis explaining elevated TSH in patients on TKIs points towards increased activity of type 3 deiodinase leading to increased T3 and T4 metabolism as has been demonstrated with other TKIs, including sorafenib and sunitinib [22, 23]. A third mechanism to explain the increased metabolism of thyroid hormone by TKIs has been proposed in the case of imatinib. Imatinib-induced hypothyroidism is thought to involve induction of uridine diphosphate– glucuronosyltransferases (UGTs), hepatic enzymes that increase clearance of T4 and T4. [24] Individuals with intact thyroid glands are able to compensate and increase T3 generation from T4, however, in hypothyroid patients relying on LT substitution alone, increased TSH results.

Diarrhea (the primary dose limiting toxicity in the pediatric clinical trial) may have contributed to decreased thyroid hormone absorption [12, 20]. At baseline nine children had diarrhea while 80% had grade 1–2 diarrhea [12] during the initial 2 cycles and fewer children presented diarrhea in advanced stages of the study. Patients with MEN2B may have intestinal ganglioneuromatosis, which may play a role in the altered thyroid hormone absorption. One proxy to screen for malabsorption is linear growth, however the overall improvement in linear growth while on vandetanib suggests that malabsorption of levothyroxine is not the underlying cause of altered TSH levels.

## Conclusions

Clinicians need to be aware of the potential downside of both over and undertreatment with thyroid hormone to ensure clinical stability. Compared to previously reported clinical trial in adults in which increased dosing of LT was required in 49.3% of athyreotic patients on vandetanib, 91% of athyreotic children in the present study required increased dosing of LT in order to maintain normal TSH values. Proper recognition and management of abnormal thyroid hormone levels is critical in growing children on TKIs.

## Abbreviations

TKIs: Tyrosine kinase inhibitors
LT: Levothyroxine
MTC: Medullary thyroid carcinoma
MEN 2B: Multiple Endocrine Neoplasia Type 2B
TFTs: Thyroid function tests
RET: REarranged during Transfection
CEA: Carcinoembryonic antigen
FMTC: Familial MTC.

## References

[1] Hogan AR, Zhuge Y, Perez EA, Koniaris LG, Lew JI, Sola JE (2009). Pediatric thyroid carcinoma: incidence and outcomes in 1753 patients. J Surg Res.

[2] Kaatsch P, Steliarova-Foucher E, Crocetti E, Magnani C, Spix C, Zambon P (2006). Time trends of cancer incidence in European children (1978–1997): report from the Automated Childhood Cancer Information System project. Eur J Cancer.

[3] Hundahl SA, Fleming ID, Fremgen AM, Menck HR (1998). A National Cancer Data Base report on 53,856 cases of thyroid carcinoma treated in the U.S., 1985–1995 [see commetns]. Cancer.

[4] Roman S, Lin R, Sosa JA (2006). Prognosis of medullary thyroid carcinoma: demographic, clinical, and pathologic predictors of survival in 1252 cases. Cancer.

[5] Raval MV, Sturgeon C, Bentrem DJ, Elaraj DM, Stewart AK, Winchester DJ, Ko CY, Reynolds M (2010). Influence of lymph node metastases on survival in pediatric medullary thyroid cancer. J Pediatr Surg.

[6] Rohmer V, Vidal-Trecan G, Bourdelot A, Niccoli P, Murat A, Wemeau JL, Borson-Chazot F, Schvartz C, Tabarin A, Chabre O (2011). Prognostic factors of disease-free survival after thyroidectomy in 170 young patients with a RET germline mutation: a multicenter study of the Groupe Francais d'Etude des Tumeurs Endocrines. J Clin Endocrinol Metabol.

[7] de Groot JW, Plukker JT, Wolffenbuttel BH, Wiggers T, Sluiter WJ, Links TP (2006). Determinants of life expectancy in medullary thyroid cancer: age does not matter. Clin Endocrinol (Oxf).

[8] Karras S, Anagnostis P, Krassas GE (2014). Vandetanib for the treatment of thyroid cancer: an update. Expert Opin Drug Metab Toxicol.

[9] Tolmachev V, Stone-Elander S, Orlova A (2010). Radiolabelled receptor-tyrosine-kinase targeting drugs for patient stratification and monitoring of therapy response: prospects and pitfalls. Lancet Oncol.

[10] Carlomagno F, Guida T, Anaganti S, Vecchio G, Fusco A, Ryan AJ, Billaud M, Santoro M (2004). Disease associated mutations at valine 804 in the RET receptor tyrosine kinase confer resistance to selective kinase inhibitors. Oncogene.

[11] Carlomagno F, Guida T, Anaganti S, Provitera L, Kjaer S, McDonald NQ, Ryan AJ, Santoro M (2009). Identification of tyrosine 806 as a molecular determinant of RET kinase sensitivity to ZD6474. Endocr Relat Cancer.

[12] Fox E, Widemann BC, Chuk MK, Marcus L, Aikin A, Whitcomb PO, Merino MJ, Lodish M, Dombi E, Steinberg SM (2013). Vandetanib in Children and Adolescents with Multiple Endocrine Neoplasia Type 2B Associated Medullary Thyroid Carcinoma. Clin Cancer Res.

[13] Brown RL (2011). Tyrosine kinase inhibitor-induced hypothyroidism: incidence, etiology, and management. Target Oncol.

[14] Wells SA, Robinson BG, Gagel RF, Dralle H, Fagin JA, Santoro M, Baudin E, Elisei R, Jarzab B, Vasselli JR (2012). Vandetanib in patients with locally advanced or metastatic medullary thyroid cancer: a randomized, double-blind phase III trial. J Clin Oncol.

[15] Brassard M, Neraud B, Trabado S, Salenave S, Brailly-Tabard S, Borget I, Baudin E, Leboulleux S, Chanson P, Schlumberger M, Young J (2011). Endocrine effects of the tyrosine kinase inhibitor vandetanib in patients treated for thyroid cancer. J Clin Endocrinol Metab.

[16] Lodish MB (2013). Clinical review: kinase inhibitors: adverse effects related to the endocrine system. J Clin Endocrinol Metab.

[17] Torino F, Corsello SM, Longo R, Barnabei A, Gasparini G (2009). Hypothyroidism related to tyrosine kinase inhibitors: an emerging toxic effect of targeted therapy. Nat Rev Clin Oncol.

[18] Makita N, Iiri T (2013). Tyrosine kinase inhibitor-induced thyroid disorders: a review and hypothesis. Thyroid.

[19] Robinson BG, Paz-Ares L, Krebs A, Vasselli J, Haddad R (2010). Vandetanib (100 mg) in patients with locally advanced or metastatic hereditary medullary thyroid cancer. J Clin Endocrinol Metabol.

[20] Brassard M, Rondeau G (2012). Role of vandetanib in the management of medullary thyroid cancer. Biologics.

[21] Ohba K, Takayama T, Matsunaga H, Matsushita A, Sasaki S, Oki Y, Ozono S, Nakamura H (2013). Inappropriate elevation of serum thyrotropin levels in patients treated with axitinib. Thyroid.

[22] Kappers MH, van Esch JH, Smedts FM, de Krijger RR, Eechoute K, Mathijssen RH, Sleijfer S, Leijten F, Danser AH, van den Meiracker AH, Visser TJ (2011). Sunitinib-induced hypothyroidism is due to induction of type 3 deiodinase activity and thyroidal capillary regression. J Clin Endocrinol Metabol.

[23] Abdulrahman RM, Verloop H, Hoftijzer H, Verburg E, Hovens GC, Corssmit EP, Reiners C, Gelderblom H, Pereira AM, Kapiteijn E (2010). Sorafenib-induced hypothyroidism is associated with increased type 3 deiodination. J Clin Endocrinol Metabol.

[24] De Groot J, Zonnenberg B, Plukker J, Van Der Graaf W, Links T (2005). Imatinib induces hypothyroidism in patients receiving levothyroxine. Clin Pharmacol Ther.

